# Complete Genome Sequence of Loktanella vestfoldensis Strain SMR4r, a Novel Strain Isolated from a Culture of the Chain-Forming Diatom Skeletonema marinoi

**DOI:** 10.1128/genomeA.01558-17

**Published:** 2018-03-22

**Authors:** Mats Töpel, Matthew I. M. Pinder, Oskar N. Johansson, Olga Kourtchenko, Anna Godhe, Adrian K. Clarke

**Affiliations:** a Department of Marine Sciences, University of Gothenburg, Gothenburg, Sweden; b Gothenburg Global Biodiversity Centre, Gothenburg, Sweden; c Department of Biological and Environmental Sciences, University of Gothenburg, Gothenburg, Sweden

## Abstract

We report here the genome sequence of Loktanella vestfoldensis strain SMR4r, isolated from the marine diatom Skeletonema marinoi strain RO5AC. Its 3,987,360-bp genome consists of a circular chromosome and two circular plasmids, one of which appears to be shared with an S. marinoi-associated Roseovarius species.

## GENOME ANNOUNCEMENT

The chain-forming diatom Skeletonema marinoi strain RO5AC associates with a large number of bacterial species, and efforts are ongoing to determine their roles in the diatom-microbe holobiont. Loktanella vestfoldensis strain SMR4r is one such bacterium which has been found as a result. The diatom strain was established from a germinated resting cell embedded in top-layer sediment, collected from 14 m depth with a box corer in May 2010 from Öresund, Sweden (55°59.16′N, 12°44.02′E). The bacterial genome was sequenced using PacBio RS II technology (Pacific Biosciences, Menlo Park, CA, USA) on one single-molecule real-time (SMRT) cell, producing 81,585 uncorrected reads totaling 845.4 Mbp. Falcon version 1.7.5 (https://github.com/PacificBiosciences/FALCON/) (1) was used to assemble the reads (seed read length, 17,100 bp), and contig circularization was confirmed by joining the corresponding ends and realigning the reads using the RS_Resequencing.1 protocol on SMRT Portal version 2.3.0 (Pacific Biosciences) (2). The final assembly contained three circular contigs totaling 3,987,360 bp (average read coverage, 177.86×).

The chromosome is 3,836,950 bp long, with a G+C content of 60.8%; plasmid pSMR4r-1 is 111,030 bp (G+C content, 57.4%), and plasmid pSMR4r-2 is 39,380 bp (G+C content, 58.2%). Strain SMR4r’s two identical 16S rRNA sequences have 99.8% identity with the three 16S sequences of L. vestfoldensis strain DSM 16212^T^ (GenBank accession number NZ_ARNL00000000). A phylotaxonomic analysis using PhyloPhlAn version 0.99 (3), comparing strain SMR4r to all whole-genome-sequenced Rhodobacteraceae strains on NCBI’s RefSeq site (ftp://ftp.ncbi.nlm.nih.gov/genomes/refseq/bacteria/), showed it to be a sister to L. vestfoldensis strain DSM 16212^T^ (100% bootstrap support). Annotation using Prokka version 1.12beta (4) predicted 3,936 coding sequences (CDSs; with 3,267 proteins having a functional prediction and 669 labeled as hypothetical), 8 pseudogenes, 45 tRNAs, 6 rRNAs, 19 noncoding RNAs (ncRNAs), and 1 transfer-messenger RNA (tmRNA).

Loktanella vestfoldensis strain SMR4r contains genes for both phosphatidylcholine synthase (LOKVESSMR4R_00456) and acyl-homoserine-lactone synthase (LOKVESSMR4R_01736, involved in quorum sensing), suggesting interaction with a eukaryote (reviewed in references 5 and 6). Loktanella vestfoldensis strain SMR4r may also be able to digest dimethylsulfoniopropionate (DMSP), an organosulfur compound produced by many phytoplankton and used by some bacteria (7); when the annotated genome of strain SMR4r was examined in Pathway Tools version 20.5 (8), three of the five enzymes in the DMSP degradation superpathway were found, two of which, *dmdA* (LOKVESSMR4R_00811) and *dmdC* (LOKVESSMR4R_00511 and LOKVESSMR4R_02869), are noted by Pathway Tools as being unique to this pathway.

There is also evidence that strain SMR4r interacts with another S. marinoi-associated bacterial species. Plasmid pSMR4r-2 contains interrupted regions accounting for the entirety of the Roseovarius mucosus strain SMR3 plasmid pSMR3-2 (GenBank accession number CP020476), with 100% sequence identity (9). These additional regions in pSMR4r-2 contain three transposase genes, two DNA invertase genes, and an additional mercuric reductase gene.

### Accession number(s).

This whole-genome project has been deposited in GenBank under the accession numbers CP021431, CP021432, and CP021433, as part of BioProject number PRJNA380207.
